# Clinical Validation of Pupil Response During Walking in Parkinson’s Disease

**DOI:** 10.3390/s26123711

**Published:** 2026-06-11

**Authors:** Lisa Graham, Rodrigo Vitorio, Alan Godfrey, Martina Mancini, Rosie Morris, Samuel Stuart

**Affiliations:** 1 Department of Sport, Exercise and Rehabilitation, Northumbria University, Newcastle NE1 8ST, UK; lisagraham722@gmail.com (L.G.); rodrigo.vitorio@northumbria.ac.uk (R.V.); rosie.e.morris@northumbria.ac.uk (R.M.); 2 Department of Computer and Information Sciences, Northumbria University, Newcastle NE1 8ST, UK; alan.godfrey@northumbria.ac.uk; 3 Department of Neurology, School of Medicine, Oregon Health & Science University, Portland, OR 97239, USA; mancinim@ohsu.edu; 4 Northumbria Healthcare NHS Foundation Trust, North Shields NE27 0QJ, UK

**Keywords:** Parkinson’s disease, pupil response, mobile eye-tracking, gait, walking, cognitive load, clinical validation, digital biomarkers, eye movement

## Abstract

**Highlights:**

**What are the main findings?**

- Pupil response during walking differentiates people with Parkinson’s disease from healthy controls, with larger pupil size and slower velocity in PD.
- Pupil response correlates with cognitive function, visual acuity, disease severity, and gait characteristics in both groups.

**What are the implications of the main findings?**

- Mobile eye-tracking during walking provides clinically valid pupil response measures that could serve as biomarkers for motor and cognitive function in PD.
- Pupil response during functional activities captures cognitive load demands that static assessments may miss.

**Abstract:**

Pupil response may be a useful biomarker in Parkinson’s disease (PD) due to links with autonomic function and cognitive load. However, research has focused on static tasks, missing functional demands during real-world activities like walking. Methods: We recruited 38 people with PD and 16 healthy controls who walked for 2 min under single- and dual-task conditions while wearing mobile eye-tracking glasses (Tobii Pro Glasses 2, 100 Hz). Pupil response outcomes (velocity, size, difference between eyes) were extracted alongside gait characteristics from inertial sensors. Known groups validity compared PD and controls; convergent/divergent validity examined relationships with cognitive, visual, clinical, and gait measures. Results: People with PD had significantly altered pupil constriction/dilation velocity (*p* = 0.01), a larger difference between their left and right pupils (*p* = 0.04), and a larger mean and minimum pupil size (*p* ≤ 0.01) compared to controls during walking. Pupil response correlated with cognitive function (JLO, CLOX1, TMTB), visual acuity, disease severity (MDS-UPDRS-III), and gait characteristics in both groups. No dual-task effects were observed. Conclusions: Pupil response during walking demonstrates known groups and convergent validity, indicating potential as a clinical biomarker for PD. Following this initial study, more research is required to further validate pupil response in PD (e.g., analytical validation and testing within real-world ecologically valid environments).

## 1. Introduction

Parkinson’s disease (PD) is a progressive neurodegenerative disease that affects motor and non-motor functioning [1]. People with PD (PwPD) experience symptoms such as tremor, rigidity, bradykinesia, and cognitive deficits [2,3,4]. The primary pathology of the disease involves dopaminergic depletion in the substantia nigra; however, there is growing research demonstrating the multifaceted nature of PD involving complex changes in several brain areas [5]. Due to the variability associated with PD, there is a definite need for reliable biomarkers that can aid in the understanding of the underlying mechanisms involved. This knowledge could aid early diagnosis, disease monitoring, and assessment of treatment efficacy. There is increasing research surrounding the potential of eye movements as biomarkers for neurological functioning, as eye movements are classically considered a window to the brain [6]. Typically, research centers around observation of saccadic and smooth pursuit eye movements. For example, anti-saccades and memory-guided saccades are known to be able to differentiate between individuals with PD and controls [7]. Subtle saccadic changes are present at the early stages of the disease [8], and changes in saccade performance have even been shown to differentiate between PD dementia and dementia with Lewy bodies [5]. Smooth pursuit eye movements are also commonly impaired in PD, with reduced gain and saccadic intrusions during tracking observed across disease stages [9]. In addition to differentiating PD from controls, eye movements have also been shown to relate to specific motor features of PD. For example, saccadic latency and variability have been shown to be increased in people with PD who experience freezing of gait (FOG) compared to those without [10], and more recently, anti-saccade abnormalities have been shown to predict FOG onset over a two-year follow-up period [11]. These findings are important for the understanding of the subtle neural differences between clinical cohorts and highlight the potential use of eye movements as biomarkers.

Pupil response (i.e., change in pupil size/diameter to varying stimuli) may also be a useful biomarker in PD due to its link to the underlying deficits in the autonomic nervous system (i.e., arousal, emotional processing, light adaptation [12], and cognitive processing [13]). It is also thought that pupil response reflects cognitive load (how much attentional and working memory resources are required to complete a task) [14]. Recent research has shown that pupil size/diameter is a useful indicator for cognitive load across different task complexities in healthy individuals [15] and PwPD [16], which may be linked to the underpinning cholinergic neural pathways that control pupil size [13]. Using pupil light reflex to assess cholinergic deficiency in PwPD and Alzheimer’s disease (AD), previous research has also shown that cholinergic dysfunction, as indicated by pupil response, is significantly associated with cognitive impairment in both PD and AD, thus suggesting that pupil response parameters are sensitive indicators of cognitive decline linked to cholinergic deficiency [17]. The main reason for observing pupil light reflex is down to acetylcholine being the main neurotransmitter involved in the dilation and constriction of the pupil’s sphincter muscle; it has been shown to be a sensitive measure for cholinergic changes [18]. The existing research on pupil response in PD is limited; however, it has been shown that a significant acetylcholine deficit in PD is reflected in the pupil light reflex [19]. More recently, there is increasing evidence for the role of serotonin in the control of pupil response. It is thought that phasic activation of serotonin neurons in the dorsal raphe nucleus within the brainstem induces transient changes in pupil size and is linked to arousal [20]. It is thought that the sympathetic nervous system controls pupil dilation and is governed by noradrenaline, and the parasympathetic nervous system causes pupil constriction via neurotransmission of catecholamines and acetylcholine [21]. Dilation controlled by the sympathetic system involves both cortical and sub-cortical domains such as the frontal cortex, hypothalamus and locus coeruleus [22]. These regions are modulated by acetylcholine and norepinephrine and are involved in aspects of cognition and attention, which is why the pupil responds not only to light stimulus but also to cognitive factors like attention and load [23]. These systems are implicated in the pathology of PD, which is why differences in pupil response are expected compared to controls [24].

At present, pupil response (activity, size, etc.) in PD is centered around seated static tasks that typically use table-top eye-tracking devices with simulated flashes on screens. For example, peak pupil constriction velocity was shown to be decreased in PD compared to controls using a static eye-tracking device [25]. Table-top eye trackers, whilst very insightful, are limited in the use of constrained and complicated eye-movement tasks, which may not be easily understood by those with neurological or cognitive impairment, such as PD. Critically, these static assessments cannot capture pupil changes during functional activities where motor and cognitive demands interact. More recently, modern mobile eye-tracking devices now allow for the recording of pupil response during naturalistic tasks that represent real-world function. Despite this, pupil response has not been studied or validated in those with PD. This represents a distinct gap in the knowledge, as cognitive load and autonomic demand may fundamentally differ during dynamic movement when compared to static tasks. Mobile devices for tracking pupil size during gait offer the potential to provide novel mechanistic insights.

This exploratory study aimed to clinically validate the measurement of pupil response via mobile eye-tracking in PD through: (1) Known Groups Validity—examine pupil response during walking under single- and dual-task conditions in PD and HC; and (2) Convergent/Divergent Validity—investigate the relationship between pupil response and other relevant measures in PD and HC. We hypothesized that pupil response would be clinically valid, specifically differentiating PD from controls and correlating with relevant clinical outcome assessments.

## 2. Materials and Methods

### 2.1. Participants

We recruited 38 PwPD and 16 healthy controls (HC) from the Oregon Health and Science University (OHSU, Portland, OR, USA) Movement Disorders Clinic. The study was weighted toward recruitment of PwPD as the primary aim was clinical validation within this population; HC were included as a normative reference group. PwPD were included if they had a clinical diagnosis of idiopathic PD by a movement disorders specialist according to the UK Brain Bank criteria, had adequate vision and hearing (Snellen chart visual acuity ≥ 12/18), and were able to walk and stand unaided. PwPD and HC were excluded if they had cognitive impairment (Montreal Cognitive Assessment, MoCA score < 21), psychiatric comorbidity, or rheumatic or orthopedic diseases that affect balance or gait, or if they were unable to comply with protocol. Informed consent was gained prior to any research activity. Ethics approval was obtained from the institutional review board at OHSU (REF: 9903).

Each participant attended a 2 h session in a controlled clinical environment at the OHSU laboratory. PwPD were tested in their usual ON medication state (within 60 min of taking anti-Parkinsonian medications). Lighting conditions were kept consistent through covering all windows and setting room lighting to the maximum output level.

### 2.2. Neuro-Cognitive Assessment(s)

Each participant was interviewed to collect basic demographic information including age (years), disease duration (years) and medical history—anti-Parkinson’s medications, history of head injury, and presence of co-morbidities, e.g., depression. The participant then underwent a series of cognitive, visual, and motor assessments with a member of the research team.

Global cognition was assessed with the MoCA (scored 0–30) [26], followed by measuring attention/executive function using Royall’s clock drawing CLOX 1 (scored 0–15) [27]. Working memory was measured through the seated forward digit span. Visuo-spatial ability was measured with Royall’s clock drawing CLOX 2 (scored 0–15) [27] and judgment of line orientation (JLO) tasks (scored 0–30) [28]. Attention and processing speed were assessed using Trail Making Test A (TMTA, seconds), and executive function and cognitive flexibility using Trail Making Test B (TMTB, seconds) [29].

Basic visual functions of acuity and contrast sensitivity were assessed using standardized charts (logMAR and logCS, respectively). Disease severity was measured with the Movement Disorder Society Unified Parkinson’s Disease Rating Scale Motor Subscale (MDS-UPDRS-III, scored out of 132) and the Hoehn and Yahr scale (Stages 0–5) [30].

### 2.3. Instrumentation

Eye tracking: A head-mounted infrared mobile eye-tracking system (Tobii Pro Glasses 2, Tobii AB., Danderyd, Sweden) recorded participant pupil size/diameter at a sample rate of 100 Hertz (Hz) during walking. Participant pupil size/diameter was recorded binocularly by four infrared illumination eye cameras. The eye-tracker was calibrated prior to data collection using the manufacturer’s single-point calibration method. Eye-tracker pupil diameter data were extracted and filtered with a moving median filter, similar to our previous mobile eye-tracking research, to remove high-frequency noise that may be introduced by artifacts (e.g., device slippage or head movements) [31].

Gait: Portable, light-weight inertial (accelerometer, gyroscope) measurement units (Opals, APDM, Portland, OR, USA) were placed on the participant at various locations (i.e., feet, lower back, chest, wrists) using elasticated Velcro straps. These inertial sensors have a high sampling rate (128 Hz) and are small in size (~44 mm × 50 mm × 14 mm (L × W × H)). Raw inertial data are streamed to APDM Mobility Lab (version 2) algorithms to provide gait outcomes including stride length, step time variability, and gait speed. This system has been validated against a pressure sensor walkway (GAITRite) in PD and healthy adults, demonstrating good-to-excellent agreement for gait velocity (ICC = 0.92 in PD), stride length (ICC = 0.88 in PD), stride time (ICC = 0.99 in PD), and stride length variability (ICC = 0.91 in PD) [32].

### 2.4. Procedure

Participants wore the mobile eye-tracker and walked, at a self-selected comfortable pace, over a 10 m straight path (with a 180° turn at each end) for 2 min (back and forth) under single- and then dual-task conditions (forward digit span, set to maximum length achieved in sitting).

The primary outcome was pupil response (left and right eye) during walking, which included average pupil constriction/dilation velocity, maximum pupil size, minimum pupil size, difference in size (between left and right), and mean pupil size. These metrics were extracted from pupil data collected with the glasses. Pupil constriction/dilation velocity was converted from millimeters/second to degrees/second to normalize measurements across individuals by accounting for variation in baseline pupil size and camera-to-eye geometry inherent to the head-mounted mobile eye-tracking device (i.e., ensuring velocity represents pupillary light reflex response rather than variation in resting pupil diameter) [33,34,35]. Additionally, conversion to degree/second ensures consistency with existing visual function literature that uses angular units (e.g., saccade velocity), which we have used in previous clinical eye-tracking studies [31,36,37]. Left and right eye outcomes were retained separately to allow detection of inter-eye asymmetry, consistent with emerging evidence that asymmetric dopaminergic degeneration in PD may be associated with inter-eye differences in pupillary function [38].

### 2.5. Statistical Analysis

Data were analyzed using SPSS (v28, IBM Inc, Chicago, IL, USA). Normal data distribution was determined using the Kolmogorov–Smirnov test, and variables were transformed where appropriate. Variables violating normality assumptions were transformed prior to inferential analyses where appropriate, including square-root transformation for maximum pupil size variables, inverse transformation for pupil velocity, and log10 transformation for left–right pupil size difference. Raw descriptive values are presented in tables for interpretability. Demographics and clinical characteristics were reported using descriptive statistics (e.g., mean, standard deviation). Between-group comparisons were conducted using appropriate parametric tests based on data distribution. To determine the validity of known groups, linear mixed effect models were conducted for each pupil response measure to examine the effect of condition (single or dual task) with group (PD, HC) as a between-subject factor. To determine the convergent/divergent validity of pupil response, we conducted Pearson’s correlations between pupil-response outcomes and other relevant demographic, cognitive, visual, clinical and gait measures. Due to the exploratory nature of this study, we did not control for multiple comparisons, and the significance level was set to *p* < 0.05 for statistical interpretation.

## 3. Results

### 3.1. Participant Demographics

Table 1 shows the participant characteristics for demographics, cognition, working memory, visual function and clinical measures. On average, PwPD were aged 68.32 years (SD = 6.69), and HC were aged 69.38 years (SD = 8.21). On average, PwPD scored 27.39/30 (SD = 2.62), and HC scored 27.63/30 (SD = 1.75) in the MoCA, indicating that both groups had intact cognition. The average disease duration for the PD group was 7.38 years (SD = 5.36), and the average MDS-UPDRS-III score was 30.71 (SD = 13.26). Gait speed, stride length and foot strike angle were significantly lower for PwPD than HC. However, there was no significant impact of dual task on gait in either group. Additionally, there was no significant difference between the groups for cognitive task error during walking (Table 1).

### 3.2. Known Groups Validation

Table 2 shows the linear mixed effect models for pupil response and gait whilst walking under single- and dual-task conditions between PD and HC. PwPD had significantly altered pupil constriction/dilation velocity (*p* = 0.01), a larger difference in size between the left and right pupils (*p* = 0.04), and larger mean and minimum pupil sizes (both left and right eyes) during walking compared to HC (all *p* ≤ 0.01). However, there was no significant change in pupil response under dual-task conditions for either group.

### 3.3. Convergent/Divergent Validity

Table 3 and Table 4 show the relationships between pupil response outcomes and cognitive, visual, clinical and gait characteristics for PwPD and HC. In PD, pupil response outcomes under single and dual tasks were significantly related to cognitive (single task: JLO (r = 0.41, *p* = 0.01); dual task: CLOX1 (r = 0.32, *p* = 0.05), JLO (r = 0.37, *p* = 0.02)), visual (single task: visual acuity (r = 0.35, *p* = 0.03)), clinical (single task: MDS-UPDRS-III (r = −0.35, *p* = 0.03)) and motor (gait) (single task: stride length (r = 0.34, *p* = 0.04); dual task: stride length (r = −0.39, *p* = 0.02)) outcomes (all moderate relationships). Therefore, worse pupil response (i.e., smaller pupils or slower pupil constriction/dilation) during walking is related to worse cognitive, visual, clinical and motor performance in PD.

In HC, pupil response outcomes under single and dual tasks were significantly related to cognitive (single task: TMTB (r = −0.62, *p* = 0.01), JLO (r = 0.55, *p* = 0.01); dual task: TMTB (r = −0.56, *p* = 0.02), JLO (r = −0.54, *p* = 0.03)) and motor (gait) (single task: foot strike angle (r = −0.51, *p* = 0.04), stride time (r = 0.50, *p* = 0.05) (both strong and moderate relationships); dual task (all strong relationships): foot strike angle (r = −0.65, *p* = 0.01, r = −0.59, *p* = 0.02)) outcomes (Table 4). Therefore, worse pupil response (i.e., smaller pupils or slower pupil constriction/dilation) during walking was related to worse cognitive and motor performance in HC.

## 4. Discussion

Literature discussing pupil response in PD is limited and typically reports static, seated tasks. This exploratory study aimed to clinically validate the measurement of pupil response via mobile eye-tracking in PD through known groups validity and convergent/divergent validity. We hypothesized that pupil response would be clinically valid, specifically differentiating PD from controls and correlating with relevant clinical outcome assessments.

### 4.1. Pupil Response During Walking Can Differentiate PD from Controls

Known groups validity was shown through pupil constriction/dilation velocity and pupil size measures (average, minimum and difference between left and right pupil size) being able to differentiate PwPD from HC, with larger pupil size and larger difference between pupil sizes (left vs. right eye) seen in PwPD compared to HC. Larger pupil size is associated with increased cognitive load [12,15,16,39], which may suggest that PwPD are functioning at higher cognitive capacity compared to HC whilst walking, irrespective of task condition. It may also be possible that the larger pupil size seen in PD is a manifestation of dopamine and norepinephrine depletion within the substantia nigra and locus coeruleus, respectively [40,41]. This is a known pathological feature of PD, and both are implicated in the neural control of pupil dilation [42]. Previous research has implicated the autonomic nervous system in the modulation of pupil size during cognitively demanding tasks in both PD and HC [15]. The sphincter muscles responsible for pupil dilation and constriction are controlled by the sympathetic and parasympathetic nervous systems via neurotransmission of catecholamines and acetylcholine, respectively [21].

Pupil sizes were not responsive to dual task whilst walking in PwPD or HC. Dual tasking is governed by higher-order cognition such as executive function and attention [39], and dual task deficiency is a known feature of PD and is associated with increased falls risk [43,44]. PwPD had larger pupil size than HC across single- and dual-task conditions, but both groups did not significantly change pupil size with the additional dual task. Lack of change in pupil size under dual task conditions may indicate that either the groups are already using cognitive resources under the single-task condition (just walking) and thus there is a ceiling effect under dual task (walking and forward digit span) [45], or they may not need to adjust their pupil size in response to a secondary cognitive task (i.e., HCs may have enough cognitive resources to deal with additional cognitive load and therefore do not adjust pupil size, or the secondary task is not challenging enough). Previous research has shown that dual-task interference is similar for PwPD and HC whilst walking and that perhaps the dual-task interference that occurs when walking is a result of the increased reliance on executive function to compensate for age-related deterioration in walking ability [46].

The gait characteristics assessed in this study were also able to differentiate PwPD from HC; however, similar to pupil size, the dual task had no influence on gait parameters in either group. PwPD had reduced gait speed, stride length, and foot strike angle compared to HC across single and dual-task conditions. This indicates that the dual task may not have been difficult enough to elicit any changes in both groups, which has been shown in previous research where digit span did not impact gait as much as other dual tasks (e.g., serial 7 s). Previous work has suggested that a dual task, such as the one used here, does not challenge the cognitive system impaired in PD as much because it is titrated to the individual’s cognitive capacity. Using a dual task such as serial 7 s is perhaps more useful to elicit deficits as it is the same task for each person and may target the cognitive areas affected by PD [46]. The lack of challenge in the secondary cognitive task is reflected in the lack of significant difference in dual-task response (% error in the cognitive task) between PwPD and HCs.

### 4.2. Pupil Response During Walking Related to Relevant Outcomes in PD and Controls

Cognitive impairment is a prominent feature of PD, and this has links to eye movement outcomes [17]. Previous research has shown that PwPD have reduced peak pupil constriction amplitude [12,47], and pupil response may be an indicator of early cognitive decline and acetylcholine impairment [17]. The current study found a relationship between increased pupil size whilst walking and better executive function in HCs (TMTB) and PwPD (CLOX1). This is not surprising, as pupil size is underpinned by cholinergic activity, which is also linked to executive-attentional function [48]. Eye movement control is also linked to other neurotransmitters that are involved in cognitive function, such as dopamine, norepinephrine, and serotonin [49]. For example, norepinephrine deficiency impacts set-shifting ability, which is assessed in the TMTB [49]. Similarly, pupil response is also related to visuo-spatial skill (JLO) in HCs and PwPD, and better visuo-spatial skill is related to larger pupil size. This relationship also suggests pupil response may be underpinned by cholinergic activity in PwPD and HCs, as acetylcholine has been found to influence visuo-spatial functioning [50].

A relationship was found between pupil size during walking and visual acuity in PwPD but not HCs, with smaller pupil size related to poorer visual acuity. Visual acuity impairment in PwPD may impact pupil response during walking, which may place them at risk of impaired mobility or even falls. Pupil size is also related to disease severity (MDS-UPDRS-III) in PwPD, with greater disease severity linked to smaller pupil size. Similarly, gait characteristics were found to relate to pupil response during walking in HC and PwPD, with larger pupil size associated with better gait under both single- and dual-task conditions. Due to the links between pupil size/response and cognitive load [14], findings suggest that walking requires cognitive resources in HC and PwPD, and also that pupil response during walking could be a potential biomarker for motor function or disease severity in PwPD. However, while we found some selective relationships for pupil size with cognition, visual function, PD severity and gait, there is a need to establish these findings in larger cohorts.

### 4.3. Study Limitations

While the research presented is the first to assess pupil response parameters in PwPD and HCs during gait, there were no changes between single- and dual-task walking. Future studies should consider alternative secondary tasks that may be more challenging for participants (e.g., serial 7 s). Additionally, the PD cohort predominantly included individuals with H&Y stage II, which may have influenced the lack of dual-task responsiveness observed in pupil response measures. Although participants demonstrated clinically relevant gait impairment and freezing of gait symptom burden, global cognition was relatively preserved, which may indicate sufficient compensatory cognitive or autonomic reserve to manage the additional dual-task demands used in this study. Future research should investigate pupil response across a broader range of disease severities, as more advanced PD may demonstrate greater alterations in pupil response during cognitively demanding walking tasks. While this research was performed in a controlled laboratory environment and precautions for lighting conditions were used, future studies should consider formal measurement of room illumination to examine the impacts of different illumination settings on pupil size and explore pupil response whilst walking in a real-world environment to increase the generalizability of findings.

Data were collected with participants ON (within 60 min of intake) their dopaminergic medication, and further testing in the OFF state is recommended to assess the role of dopamine on pupil response. Similarly, this study did not control for other anti-Parkinsonian or other medications that may influence pupil response (e.g., antidepressants) and future studies should consider the effect of anti-cholinergic medication on pupil response parameters. Lastly, this study did not control for sleepiness or time of day, and future work should consider these factors. However, previous work suggests that baseline pupil diameter may not represent a consistent or reliable biomarker of subjective sleepiness across all times of day [51].

## 5. Conclusions

This was the first study to assess pupil response while walking in PwPD and HCs via mobile eye-tracking. This exploratory study has shown that pupil response parameters during walking have known groups validity and convergent validity, which indicates that these pupil response outcomes could be used within clinical assessments. Further work is required to establish findings in larger cohorts to examine the pupil response measures in relation to interventions (i.e., medications).

## Figures and Tables

**Table 1:** Demographic, cognitive, visual, and clinical characteristics.

	Parkinson’s Disease (PD) Mean (SD)	Healthy Controls (HC) Mean (SD)	*p*
***Demographics***			
Age (years)	68.32 (6.69)	69.38 (8.21)	0.622
Sex	25 M/13 F	9 M/7 F	-
Height (meters)	1.73 (0.11)	1.71 (0.10)	0.589
Weight (lbs.)	180.87 (43.46)	167.13 (29.73)	0.254
Education (years)	16.84 (2.74)	17.69 (2.73)	0.304
GDS-15	6.16 (4.87)	3.00 (4.29)	0.029
FES-I	27.29 (10.20)	18.56 (1.93)	0.001
***Global Cognition***			
MoCA	27.39 (2.62)	27.63 (1.75)	0.749
Attention/Executive function			
CLOX 1	12.71 (1.71)	12.13 (1.50)	0.403
TMTB	82.51 (59.97)	55.38 (18.58)	0.083
Visuo-spatial ability			
CLOX 2	14.00 (1.03)	13.50 (1.51)	0.166
TMTA	34.58 (18.10)	22.23 (6.28)	0.010
JLO	24.03 (6.90)	27.50 (2.45)	0.056
Working memory			
Forward Digit span (sitting)	6.05 (1.18)	6.06 (1.00)	0.977
***Visual function***			
Visual acuity (binocular)	0.07 (0.15)	0.04 (0.14)	0.483
Contrast sensitivity (binocular)	1.57 (0.18)	1.66 (0.13)	0.048
***Clinical***			
Disease Duration (Years)	7.38 (5.36)	-	-
LEDD	774.70 (406.17)	-	-
MDS-UPDRS-III	30.71 (13.26)	-	-
H&Y	34 II/4 III	-	-
FOG-Q	7.45 (9.61)	-	-
Number of Freezers	18 FOG/20 nFOG	-	-
Dual-task cognitive			
Walk (% error)	31.21 (18.78)	23.12 (20.25)	0.163

[M = male, F = female, MoCA = Montreal Cognitive Assessment, GDS-15 = Geriatric Depression Scale-15, FES-I = Falls Efficacy Scale—International, SD = standard deviation, *p* = *p*-value, significance level, LEDD = levodopa equivalent daily dose, MDS-UPDRS-III = Unified Parkinson’s Disease Rating Scale III, H&Y = Hoehn & Yahr stage, FOG-Q = freezing of gait questionnaire].

**Table 2:** Linear mixed effects model results for pupil response whilst walking during single and dual tasks in PwPD and HC.

	Single Task (ST)		Dual Task (DT)		Linear Mixed Effect Model		
Pupil Response Variable	PD	HC	PD	HC	Task (ST, DT) *p*	Group (HC, PD) *p*	Task × Group *p*
Average Pupil Constriction/Dilation Velocity (°/s)	12.58 (5.82)	9.33 (3.44)	14.25 (7.12)	11.08 (4.58)	0.17	**0.01**	0.97
Max Pupil Size Left (mm)	6.27 (1.29)	5.93 (1.05)	6.14 (1.24)	6.30 (0.82)	0.63	0.72	0.31
Max Pupil Size Right (mm)	6.04 (1.10)	5.88 (0.98)	6.08 (1.24)	6.04 (0.86)	0.68	0.68	0.79
Difference in Size (Left vs. Right) (mm)	0.21 (0.13)	0.16 (0.14)	0.25 (0.18)	0.19 (0.15)	0.58	**0.04**	0.99
Mean Pupil Size Left (mm)	3.73 (0.50)	3.40 (0.52)	3.86 (0.54)	3.57 (0.56)	0.18	**0.01**	0.86
Mean Pupil Size Right (mm)	3.73 (0.49)	3.34 (0.45)	3.89 (0.53)	3.49 (0.48)	0.14	**<0.001**	0.99
Min Pupil Size Left (mm)	2.64 (0.39)	2.40 (0.34)	2.75 (0.38)	2.42 (0.36)	0.40	**<0.001**	0.59
Min Pupil Size Right (mm)	2.65 (0.39)	2.46 (0.36)	2.68 (0.40)	2.40 (0.26)	0.90	**0.003**	0.55
Gait Characteristic							
Gait speed (m/s)	0.98 (0.17)	1.11 (0.14)	0.92 (0.18)	1.06 (0.15)	0.13	**<0.001**	0.88
Stride length (m)	1.05 (0.16)	1.18 (0.09)	1.00 (0.16)	1.14 (0.10)	0.17	**<0.001**	0.88
Stride time (s)	1.09 (0.10)	1.07 (0.09)	1.11 (0.11)	1.09 (0.10)	0.35	0.41	0.93
Foot strike angle (°)	12.59 (5.37)	17.99 (3.02)	11.61 (5.34)	17.19 (2.89)	0.38	**<0.001**	0.94

[°/s = degrees per second, mm = millimeters, m/s = meters per second, m = meters, s = seconds, ° = degrees, *p* = significance level (*p* < 0.05, highlighted in bold). Means given for each eye movement and gait outcome and standard deviations indicated in brackets. PwPD = people with PD. HC = healthy controls. PD = Parkinson’s disease. ST = single task. DT = dual task. Pupil velocity is expressed in degrees/second to standardize measurements across individuals. Raw descriptive values are shown while inferential analyses used transformed variables as described in the methodology].

**Table 3:** Correlations for PwPD between pupil response outcomes and cognitive, visual, clinical and gait measures.

PD		Cognition					Vision		Clinical				Gait			
	Age	CLOX1	CLOX2	TMTA	TMTB	JLO	VA	CS	Disease Duration	LEDD	MDS-UPDRS-III	H&Y	Gait Speed	Stride Length	Foot Strike Angle	Stride Time
Single Task																
Average Pupil Constriction/Dilation Velocity (°/s)	0.20 (0.24)	0.14 (0.39)	0.05 (0.75)	−0.21 (0.20)	−0.09 (0.57)	0.29 (0.08)	−0.25 (0.14)	0.12 (0.47)	−0.14 (0.41)	−0.19 (0.23)	−0.21 (0.21)	−0.07 (0.67)	0.01 (0.93)	0.13 (0.42)	0.10 (0.54)	0.18 (0.29)
Max Pupil Size Left (mm)	0.21 (0.21)	0.16 (0.33)	0.13 (0.43)	−0.14 (0.42)	−0.13 (0.42)	0.15 (0.38)	−0.06 (0.74)	0.13 (0.45)	−0.28 (0.09)	−0.21 (0.20)	−0.35 (0.03 *)	−0.09 (0.58)	0.26 (0.12)	0.34 (0.04) *	0.22 (0.18)	0.06 (0.74)
Max Pupil Size Right (mm)	0.19 (0.24)	0.06 (0.73)	−0.06 (0.73)	0.18 (0.28)	0.01 (0.98)	−0.01 (0.97)	0.35 (0.03) *	−0.14 (0.39)	−0.01 (0.94)	−0.01 (0.96)	0.17 (0.31)	0.02 (0.93)	0.08 (0.62)	0.16 (0.35)	−0.01 (0.99)	0.08 (0.61)
Difference in Size (Left vs. Right) (mm)	−0.003 (0.99)	0.05 (0.77)	−0.14 (0.41)	0.01 (0.97)	0.03 (0.85)	0.15 (0.39)	0.11 (0.50)	−0.01 (0.94)	0.09 (0.60)	−0.12 (0.48)	0.08 (0.63)	−0.11 (0.52)	0.002 (0.99)	−0.05 (0.79)	−0.07 (0.68)	−0.10 (0.57)
Mean Pupil Size Left (mm)	−0.12 (0.49)	0.24 (0.14)	0.02 (0.91)	0.02 (0.91)	0.14 (0.41)	0.10 (0.54)	0.09 (0.58)	0.04 (0.84)	−0.05 (0.76)	0.04 (0.82)	0.02 (0.90)	−0.03 (0.86)	−0.10 (0.54)	−0.12 (0.47)	0.08 (0.65)	−0.06 (0.72)
Mean Pupil Size Right (mm)	−0.19 (0.26)	0.31 (0.06)	0.06 (0.71)	0.05 (0.78)	0.14 (0.41)	0.14 (0.41)	0.18 (0.29)	−0.02 (0.92)	−0.04 (0.81)	0.17 (0.30)	0.12 (0.47)	−0.11 (0.53)	−0.06 (0.74)	−0.06 (0.73)	0.13 (0.42)	−0.07 (0.68)
Min Pupil Size Left (mm)	−0.16 (0.34)	0.25 (0.13)	0.10 (0.55)	−0.07 (0.68)	0.05 (0.75)	0.17 (0.31)	−0.09 (0.59)	0.05 (0.77)	−0.12 (0.49)	0.02 (0.89)	−0.10 (0.53)	−0.11 (0.52)	−0.02 (0.92)	0.03 (0.87)	0.13 (0.87)	0.03 (0.86)
Min Pupil Size Right (mm)	0.01 (0.99)	0.26 (0.12)	0.15 (0.36)	−0.13 (0.44)	−0.11 (0.52)	0.41 (0.01) **	−0.07 (0.69)	−0.01 (0.96)	−0.16 (0.34)	−0.05 (0.76)	−0.07 (0.70)	0.04 (0.81)	−0.01 (0.94)	0.08 (0.64)	0.18 (0.29)	0.12 (0.47)
Dual Task																
Average Pupil Constriction/Dilation Velocity (°/s)	0.12 (0.47)	0.11 (0.51)	0.05 (0.77)	−0.12 (0.48)	0.08 (0.63)	0.13 (0.43)	−0.10 (0.55)	0.12 (0.46)	−0.04 (0.83)	−0.18 (0.27)	−0.20 (0.23)	0.02 (0.89)	0.03 (0.87)	0.05 (0.75)	0.08 (0.66)	0.05 (0.77)
Max Pupil Size Left (mm)	0.26 (0.11)	0.13 (0.43)	0.16 (0.35)	−0.19 (0.25)	−0.05 (0.75)	0.19 (0.24)	−0.22 (0.19)	0.06 (0.74)	−0.17 (0.32)	−0.06 (0.71)	−0.24 (0.16)	−0.14 (0.16)	0.24 (0.16)	0.39 (0.02) *	0.26 (0.12)	0.15 (0.36)
Max Pupil Size Right (mm)	0.07 (0.67)	0.02 (0.90)	0.09 (0.60)	−0.01 (0.95)	−0.10 (0.56)	0.17 (0.30)	0.09 (0.58)	−0.15 (0.36)	−0.12 (0.48)	−0.08 (0.65)	−0.05 (0.75)	−0.19 (0.25)	0.17 (0.32)	0.26 (0.13)	0.10 (0.75)	0.04 (0.80)
Difference in Size (Left vs. Right) (mm)	−0.11 (0.50)	0.07 (0.68)	−0.10 (0.54)	0.18 (0.27)	0.15 (0.38)	0.09 (0.59)	0.08 (0.66)	−0.12 (0.51)	0.12 (0.53)	0.11 (0.51)	0.17 (0.30)	−0.18 (0.27)	−0.04 (0.80)	−0.04 (0.80)	−0.04 (0.78)	−0.09 (0.62)
Mean Pupil Size Left (mm)	−0.08 (0.64)	0.24 (0.15)	0.06 (0.73)	−0.04 (0.83)	0.04 (0.81)	0.19 (0.27)	0.03 (0.85)	0.05 (0.79)	−0.14 (0.40)	−0.04 (0.84)	−0.06 (0.70)	−0.06 (0.72)	0.04 (0.80)	0.09 (0.61)	0.21 (0.22)	−0.01 (0.94)
Mean Pupil Size Right (mm)	−0.15 (0.38)	0.32 (0.05) *	0.17 (0.30)	0.04 (0.80)	−0.08 (0.66)	0.26 (0.11)	0.07 (0.70)	−0.002 (0.99)	−0.14 (0.40)	0.12 (0.49)	0.05 (0.78)	−0.13 (0.45)	0.05 (0.78)	0.11 (0.52)	0.23 (0.16)	−0.02 (0.92)
Min Pupil Size Left (mm)	−0.06 (0.71)	0.17 (0.31)	0.03 (0.84)	0.03 (0.86)	−0.02 (0.89)	0.28 (0.10)	0.07 (0.69)	−0.07 (0.67)	−0.19 (0.26)	−0.05 (0.75)	−0.11 (0.51)	−0.05 (0.77)	0.05 (0.76)	0.09 (0.58)	0.25 (0.14)	−0.01 (0.97)
Min Pupil Size Right (mm)	0.01 (0.94)	0.25 (0.13)	0.06 (0.72)	0.05 (0.76)	−0.10 (0.54)	0.37 (0.02) **	0.12 (0.48)	−0.08 (0.65)	−0.12 (0.49)	−0.04 (0.79)	−0.12 (0.48)	−0.03 (0.85)	0.07 (0.69)	0.22 (0.19)	0.24 (0.15)	0.17 (0.31)

[°/s = degrees per second, mm = millimeters, TMTA = trail making task A, TMTB = trail making task B, JLO = judgment of line orientation task, VA = visual acuity, CS = contrast sensitivity, LEDD = levodopa equivalent daily dose, MDS-UPDRS-III = Movement Disorders Society Unified Parkinson’s Disease Rating Scale-III, H&Y = Hoehn & Yahr. Values represent Pearson’s r with *p*-values in parentheses. * = *p* < 0.05, ** = *p* < 0.01].

**Table 4:** Correlations for HC between pupil response outcomes and cognitive, visual and gait measures.

HC		Cognition					Vision		Gait			
	Age	CLOX1	CLOX2	TMTA	TMTB	JLO	VA	CS	Gait Speed	Stride Length	Foot Strike Angle	Stride Time
Single Task												
Average Pupil Constriction/Dilation Velocity (°/s)	−0.23 (0.39)	−0.13 (0.62)	0.18 (0.50)	−0.22 (0.42)	−0.16 (0.56)	0.11 (0.68)	−0.04 (0.90)	0.03 (0.92)	0.06 (0.82)	0.16 (0.56)	−0.08 (0.76)	0.02 (0.93)
Max Pupil Size Left (mm)	−0.29 (0.27)	0.21 (0.43)	0.25 (0.36)	−0.44 (0.09)	−0.62 (0.01) **	0.55 (0.01) **	0.24 (0.61)	0.02 (0.93)	−0.40 (0.12)	−0.22 (0.41)	−0.11 (0.69)	0.37 (0.16)
Max Pupil Size Right (mm)	0.22 (0.41)	0.40 (0.12)	0.21 (0.44)	0.12 (0.65)	0.12 (0.67)	−0.01 (0.97)	−0.04 (0.90)	−0.14 (0.60)	−0.39 (0.13)	−0.11 (0.69)	0.12 (0.65)	0.50 (0.05) *
Difference in Size (Left vs. Right) (mm)	0.9 (0.75)	0.38 (0.16)	−0.06 (0.82)	0.13 (0.66)	0.03 (0.93)	−0.28 (0.31)	−0.23 (0.41)	0.16 (0.56)	−0.30 (0.27)	−0.48 (0.07)	−0.48 (0.07)	0.02 (0.95)
Mean Pupil Size Left (mm)	0.35 (0.19)	−0.04 (0.90)	−0.02 (0.95)	−0.23 (0.40)	−0.49 (0.06)	0.31 (0.25)	−0.02 (0.93)	0.33 (0.22)	−0.23 (0.40)	−0.24 (0.37)	−0.51 (0.04) *	0.10 (0.71)
Mean Pupil Size Right (mm)	−0.37 (0.16)	−0.16 (0.56)	0.02 (0.95)	−0.26 (0.32)	−0.40 (0.13)	0.24 (0.37)	−0.004 (0.99)	0.39 (0.14)	−0.20 (0.46)	−0.25 (0.34)	−0.47 (0.07)	0.06 (0.84)
Min Pupil Size Left (mm)	−0.28 (0.29)	0.22 (0.41)	0.16 (0.57)	−0.07 (0.80)	−0.38 (0.14)	0.44 (0.09)	0.13 (0.63)	0.01 (0.98)	−0.19 (0.49)	−0.19 (0.49)	−0.21 (0.44)	0.19 (0.49)
Min Pupil Size Right (mm)	−0.46 (0.07)	−0.27 (0.32)	−0.13 (0.63)	−0.19 (0.48)	−0.46 (0.07)	0.57 (0.02) **	−0.06 (0.82)	0.20 (0.47)	−0.13 (0.62)	−0.01 (0.98)	−0.25 (0.35)	0.16 (0.55)
Dual Task												
Average Pupil Constriction/Dilation Velocity (°/s)	−0.32 (0.23)	0.04 (0.90)	0.34 (0.20)	−0.19 (0.47)	−0.35 (0.18)	0.13 (0.64)	0.004 (0.99)	0.05 (0.85)	0.11 (0.68)	0.28 (0.30)	−0.06 (0.84)	0.05 (0.85)
Max Pupil Size Left (mm)	−0.11 (0.69)	0.33 (0.22)	0.29 (0.28)	−0.42 (0.11)	0.35 (0.19)	0.11 (0.70)	−0.10 (0.71)	0.15 (0.58)	−0.03 (0.93)	−0.03 (0.93)	−0.21 (0.44)	−0.04 (0.88)
Max Pupil Size Right (mm)	0.28 (0.29)	0.40 (0.13)	0.06 (0.82)	0.11 (0.68)	−0.03 (0.93)	−0.09 (0.74)	−0.06 (0.82)	−0.22 (0.41)	−0.22 (0.41)	0.02 (0.41)	0.13 (0.63)	0.33 (0.21)
Difference in Size (Left vs. Right) (mm)	0.33 (0.21)	0.37 (0.16)	0.02 (0.95)	0.10 (0.71)	0.24 (0.38)	−0.22 (0.42)	−0.10 (0.71)	0.02 (0.95)	−0.16 (0.56)	−0.17 (0.52)	−0.23 (0.39)	0.11 (0.68)
Mean Pupil Size Left (mm)	−0.35 (0.19)	−0.10 (0.71)	−0.05 (0.85)	−0.24 (0.38)	−0.42 (0.11)	0.30 (0.27)	−0.11 (0.68)	0.40 (0.13)	−0.21 (0.43)	−0.17 (0.53)	−0.65 (0.01) **	0.22 (0.42)
Mean Pupil Size Right (mm)	−0.38 (0.15)	−0.23 (0.40)	0.01 (0.96)	−0.26 (0.33)	−0.30 (0.25)	0.26 (0.32)	−0.07 (0.81)	0.43 (0.09)	−0.22 (0.41)	−0.20 (0.46)	−0.59 (0.02) *	0.21 (0.44)
Min Pupil Size Left (mm)	−0.46 (0.08)	−0.06 (0.83)	0.25 (0.36)	−0.05 (0.87)	−0.38 (0.15)	0.38 (0.15)	−0.06 (0.83)	0.11 (0.68)	0.10 (0.71)	0.27 (0.31)	−0.26 (0.33)	0.10 (0.70)
Min Pupil Size Right (mm)	−0.23 (0.39)	−0.05 (0.84)	0.17 (0.54)	−0.41 (0.12)	−0.56 (0.02) *	0.54 (0.03) *	0.22 (0.42)	0.12 (0.67)	0.06 (0.82)	−0.04 (0.88)	−0.42 (0.10)	−0.12 (0.67)

[°/s = degrees per second, mm = millimeters, TMTA = trail making task A, TMTB = trail making task B, JLO = judgment of line orientation task, VA = visual acuity, CS = contrast sensitivity. Values represent Pearson’s r with *p*-values in parentheses. * = *p* < 0.05, ** = *p* < 0.01].

## Data Availability

Data are available upon reasonable request from the corresponding author.

## Abbreviations

The following abbreviations are used in this manuscript:

CLOX1	Clock Drawing Test (executive function)
CLOX2	Clock Drawing Test (copying)
CS	Contrast Sensitivity
DT	Dual Task
FES-I	Falls Efficacy Scale—International
FOG-Q	Freezing of Gait Questionnaire
GDS-15	Geriatric Depression Scale-15
H&Y	Hoehn and Yahr scale
HC	Healthy Controls
JLO	Judgment of Line Orientation
LEDD	Levodopa Equivalent Daily Dose
MoCA	Montreal Cognitive Assessment
OHSU	Oregon Health and Science University
PD	Parkinson’s Disease
ST	Single Task
TMTA	Trail Making Test Part A
TMTB	Trail Making Test Part B
MDS-UPDRS-III	Movement Disorders Society Unified Parkinson’s Disease Rating Scale Motor Subscale
VA	Visual Acuity

## References

[1] Kieburtz K., Wunderle K.B. (2013). Parkinson’s disease: Evidence for environmental risk factors. Mov. Disord..

[2] Hoehn M.M., Yahr M.D. (1998). Parkinsonism: Onset, progression, and mortality. Neurology.

[3] Postuma R.B., Berg D. (2019). Prodromal Parkinson’s Disease: The Decade Past, the Decade to Come. Mov. Disord..

[4] Stuart S., Lawson R.A., Yarnall A.J., Nell J., Alcock L., Duncan G.W., Khoo T.K., Barker R.A., Rochester L., Burn D.J. (2019). Pro-Saccades Predict Cognitive Decline in Parkinson’s Disease: ICICLE-PD. Mov. Disord..

[5] Mosimann U.P., Müri R.M., Burn D.J., Felblinger J., O’BRien J.T., McKeith I.G. (2005). Saccadic eye movement changes in Parkinson’s disease dementia and dementia with Lewy bodies. Brain.

[6] Graham L., Vitorio R., Walker R., Godfrey A., Morris R., Stuart S. (2025). Digital measurement of ocular microtremor in Parkinson’s disease: Protocol for a pilot study to assess reliability and clinical validation. PLoS ONE.

[7] Blekher T., Weaver M., Rupp J., Nichols W.C., Hui S.L., Gray J., Yee R.D., Wojcieszek J., Foroud T. (2009). Multiple step pattern as a biomarker in Parkinson disease. Park. Relat. Disord..

[8] MacAskill M.R., Graham C.F., Pitcher T.L., Myall D.J., Livingston L., van Stockum S., Dalrymple-Alford J.C., Anderson T.J. (2012). The influence of motor and cognitive impairment upon visually-guided saccades in Parkinson’s disease. Neuropsychologia.

[9] Frei K. (2021). Abnormalities of smooth pursuit in Parkinson’s disease: A systematic review. Clin. Park. Relat. Disord..

[10] Nemanich S.T., Earhart G.M. (2016). Freezing of gait is associated with increased saccade latency and variability in Parkinson’s disease. Clin. Neurophysiol..

[11] Fernandez-Ruiz J., Riek H.C., Brien D.C., Coe B.C., Grimes D.A., Lang A.E., Marras C., Masellis M., Swartz R.H., Tan B. (2025). The relation of eye movements to the occurrence of freezing of gait in Parkinson’s disease. Brain Commun..

[12] Micieli G., Tassorelli C., Martignoni E., Pacchetti C., Bruggi P., Magri M., Nappi G. (1991). Disordered pupil reactivity in Parkinson’s disease. Clin. Auton. Res..

[13] Wang C.-A., Munoz D.P. (2015). A circuit for pupil orienting responses: Implications for cognitive modulation of pupil size. Curr. Opin. Neurobiol..

[14] Vogels J., Demberg V., Kray J. (2018). The Index of Cognitive Activity as a Measure of Cognitive Processing Load in Dual Task Settings. Front. Psychol..

[15] Krejtz K., Duchowski A.T., Niedzielska A., Biele C., Krejtz I. (2018). Eye tracking cognitive load using pupil diameter and microsaccades with fixed gaze. PLoS ONE.

[16] Kahya M., Moon S., Lyons K.E., Pahwa R., Akinwuntan A.E., Devos H. (2018). Pupillary Response to Cognitive Demand in Parkinson’s Disease: A Pilot Study. Front. Aging Neurosci..

[17] Fotiou D.F., Stergiou V., Tsiptsios D., Lithari C., Nakou M., Karlovasitou A. (2009). Cholinergic deficiency in Alzheimer’s and Parkinson’s disease: Evaluation with pupillometry. Int. J. Psychophysiol..

[18] Loewenfeld I.E., Lowenstein O. (1999). The Pupil: Anatomy, Physiology, and Clinical Applications.

[19] Granholm E., Morris S., Galasko D., Shults C., Rogers E., Vukov B. (2003). Tropicamide effects on pupil size and pupillary light reflexes in Alzheimer’s and Parkinson’s disease. Int. J. Psychophysiol..

[20] Cazettes F., Reato D., Morais J.P., Renart A., Mainen Z.F. (2021). Phasic Activation of Dorsal Raphe Serotonergic Neurons Increases Pupil Size. Curr. Biol..

[21] Ajasse S., Benosman R.B., Lorenceau J. (2018). Effects of pupillary responses to luminance and attention on visual spatial discrimination. J. Vis..

[22] Tsitsi P., Benfatto M.N., Seimyr G.Ö., Larsson O., Svenningsson P., Markaki I. (2021). Fixation Duration and Pupil Size as Diagnostic Tools in Parkinson’s Disease. J. Park. Dis..

[23] Eckstein M.K., Guerra-Carrillo B., Singley A.T.M., Bunge S.A. (2017). Beyond eye gaze: What else can eyetracking reveal about cognition and cognitive development?. Dev. Cogn. Neurosci..

[24] Bari B.A., Chokshi V., Schmidt K. (2020). Locus coeruleus-norepinephrine: Basic functions and insights into Parkinson’s disease. Neural Regen. Res..

[25] Tsitsi P., Nilsson M., Waldthaler J., Seimyr G.Ö., Larsson O., Svenningsson P., Markaki I. (2023). Pupil light reflex dynamics in Parkinson’s disease. Front. Integr. Neurosci..

[26] Dalrymple-Alford J.C., MacAskill M.R., Nakas C.T., Livingston L., Graham C., Crucian G.P., Melzer T.R., Kirwan J., Keenan R., Wells S. (2010). The MoCA. Neurology.

[27] Royall D.R., Cordes J.A., Polk M. (1998). CLOX: An executive clock drawing task. J. Neurol. Neurosurg. Psychiatry.

[28] Wechsler D. (1945). A Standardized Memory Scale for Clinical Use. J. Psychol..

[29] Reitan R.M. (1958). Validity of the Trail Making Test as an indicator of organic brain damage. Percept. Mot. Ski..

[30] Goetz C.G., Fahn S., Martinez-Martin P., Poewe W., Sampaio C., Stebbins G.T., Stern M.B., Tilley B.C., Dodel R., Dubois B. (2007). Movement Disorder Society-sponsored revision of the Unified Parkinson’s Disease Rating Scale (MDS-UPDRS): Process, format, and clinimetric testing plan. Mov. Disord..

[31] Stuart S., Parrington L., Martini D., Popa B., Fino P.C., King L.A. (2019). Validation of a velocity-based algorithm to quantify saccades during walking and turning in mild traumatic brain injury and healthy controls. Physiol. Meas..

[32] Morris R., Stuart S., McBarron G., Fino P.C., Mancini M., Curtze C. (2019). Validity of Mobility Lab (version 2) for gait assessment in young adults, older adults and Parkinson’s disease. Physiol. Meas..

[33] Niehorster D.C., Hessels R.S., Benjamins J.S. (2020). GlassesViewer: Open-source software for viewing and analyzing data from the Tobii Pro Glasses 2 eye tracker. Behav. Res. Methods.

[34] Mathôt S. (2018). Pupillometry: Psychology, physiology, and function. J. Cogn..

[35] Mathôt S., Vilotijević A. (2023). Methods in cognitive pupillometry: Design, preprocessing, and statistical analysis. Behav. Res. Methods.

[36] Lirani-Silva E., Stuart S., Parrington L., Campbell K., King L. (2021). Saccade and fixation Eye movements during walking in people With mild traumatic brain injury. Front. Bioeng. Biotechnol..

[37] Graham L., Armitage J., Vitorio R., Das J., Barry G., Godfrey A., Stuart S. (2023). Visual exploration while walking with and without visual cues in Parkinson’s disease: Freezer versus non-freezer. Neurorehabilit. Neural Repair.

[38] Hansen F.O., Knudsen K., Damholdt M.F., Bek T., Borghammer P., Okkels N. (2025). Non-motor asymmetry and dopamine degeneration in Parkinson’s disease. Brain Commun..

[39] Kahya M., Lyons K.E., Pahwa R., Akinwuntan A.E., He J., Devos H. (2021). Reliability and Validity of Pupillary Response During Dual-Task Balance in Parkinson Disease. Arch. Phys. Med. Rehabil..

[40] Drew D.S., Muhammed K., Baig F., Kelly M., Saleh Y., Sarangmat N., Okai D., Hu M., Manohar S., Husain M. (2020). Dopamine and reward hypersensitivity in Parkinson’s disease with impulse control disorder. Brain.

[41] Espay A.J., LeWitt P.A., Kaufmann H. (2014). Norepinephrine deficiency in Parkinson’s disease: The case for noradrenergic enhancement. Mov. Disord..

[42] Viglione A., Mazziotti R., Pizzorusso T. (2023). From pupil to the brain: New insights for studying cortical plasticity through pupillometry. Front. Neural Circuits.

[43] Fuller R.L., Van Winkle E.P., Anderson K.E., Gruber-Baldini A.L., Hill T., Zampieri C., Weiner W.J., Shulman L.M. (2013). Dual task performance in Parkinson’s disease: A sensitive predictor of impairment and disability. Park. Relat. Disord..

[44] Heinzel S., Maechtel M., Hasmann S.E., Hobert M.A., Heger T., Berg D., Maetzler W. (2016). Motor dual-tasking deficits predict falls in Parkinson’s disease: A prospective study. Park. Relat. Disord..

[45] Baddeley A. (1992). Working Memory. Science.

[46] Rochester L., Galna B., Lord S., Burn D. (2014). The nature of dual-task interference during gait in incident Parkinson’s disease. Neuroscience.

[47] Beaumont S.M., Harris J.P., Leendertz J.A., Phillipson O.T. (1987). The pupillary light reflex in mild Parkinson’s disease. Clin. Vis. Sci..

[48] Klinkenberg I., Sambeth A., Blokland A. (2011). Acetylcholine and attention. Behav. Brain Res..

[49] Robbins T.W., Roberts A.C. (2007). Differential regulation of fronto-executive function by the monoamines and acetylcholine. Cereb. Cortex.

[50] Gratton C., Yousef S., Aarts E., Wallace D.L., D’ESposito M., Silver M.A. (2017). Cholinergic, But Not Dopaminergic or Noradrenergic, Enhancement Sharpens Visual Spatial Perception in Humans. J. Neurosci..

[51] Daguet I., Bouhassira D., Gronfier C. (2019). Baseline Pupil Diameter Is Not a Reliable Biomarker of Subjective Sleepiness. Front. Neurol..

